# Complete response of metastatic melanoma in a patient with Crohn’s disease simultaneously receiving anti-α4β7 and anti-PD1 antibodies

**DOI:** 10.1186/s40425-018-0484-x

**Published:** 2019-01-06

**Authors:** Christopher C. Frohne, Ernesto M. Llano, Ashley Perkovic, Russell D. Cohen, Jason J. Luke

**Affiliations:** 1 Department of Medicine, University of Chicago, 5841 S. Maryland Ave. MC2115, Chicago, IL 60637 USA; 2 Department of Medicine, Section of Gastroenterology, Inflammatory Bowel Disease Center, University of Chicago, Chicago, IL USA

**Keywords:** Immune checkpoint inhibitor therapy, Metastatic melanoma, Crohn’s disease, Autoimmune disease, Immune related adverse event

## Abstract

**Background:**

Immune checkpoint inhibitors (ICPIs) are increasingly being used in the treatment of a variety of malignancies. The original studies that demonstrated the efficacy of ICPIs excluded patients actively being treated for autoimmune conditions, and there is only limited evidence that these treatments are safe and effective in this population of patients.

**Case presentation:**

We present a case of a man with Crohn’s disease actively requiring immunosuppressive therapy who subsequently received pembrolizumab for metastatic melanoma. He had no further progression of metastatic disease and had resolution of his pulmonary nodule while he experienced no Crohn’s disease flares or immune related adverse events. We surveyed the existing literature for studies examining the use of ICPIs in patients with autoimmune disorders and reviewed the unique mechanism of action of the α4β7 inhibitor, vedolizumab.

**Conclusion:**

Patients with autoimmune conditions should be considered candidates for immune checkpoint inhibition even in the setting of active immunosuppressive therapy. The mechanism of action of immunosuppressive therapy should be considered with the most targeted form of treatment being used when possible. Further prospective studies investigating immunotherapy in patients with autoimmune conditions are warranted.

## Background

Immune checkpoint inhibition has rapidly changed the standard of care for a variety of malignancies. Overall survival has been improved in patients with melanoma with the anti-cytotoxic T-lymphocyte associated antigen 4 antibody ipilimumab [1] and broadly across many cancers with anti-programmed death receptor-1 (PD1)/PD-ligand-1 (PD-L1) antibodies [2]. Anti-PD-1 antibodies such as nivolumab and pembrolizumab are thought to enact their anti-cancer effects by relieving the suppression of PD-L1, a physiologic mechanism controlling activated CD8 T cells to avoid chronic autoimmune inflammation [3]. This balance of efficacy versus toxicity is evidenced by checkpoint inhibitor toxicities such as enterocolitis, hypophysitis, thyroiditis, pneumonitis, and others [2]. Immune related adverse events (irAEs) are common and depending on the severity, can require cessation of therapy as well as glucocorticoids, anti-tumor necrosis factor antibodies or other forms of immunosuppression. This toxicities spectrum raises the question of whether patients with pre-existing autoimmune conditions should be treated with this class of therapy. Clinical trials demonstrating the efficacy of checkpoint blockade excluded patients with autoimmune conditions [1, 4–7]. While there are retrospective studies that assess whether these agents can be safely used in patients with autoimmune conditions, that has not been evaluated in many clinically relevant scenarios. We present a unique case in which vedolizumab, an α4β7 integrin inhibitor that limits T cell trafficking, is used simultaneously with pembrolizumab in the successful treatment of a patient with metastatic melanoma who additionally has an active diagnosis of Crohn’s disease.

### Case presentation

A 59 year old man with Crohn’s disease presented to dermatology in March 2016 with a scalp growth. Biopsy showed a spindle cell/desmoplastic melanoma (Clark level IV, Breslow thickness 1.75 mm, mitotic figures at least 5/mm^2^, no perineural/lymphatic invasion) with positive deep margins. In April 2016 he underwent wide excision with sentinel lymph node biopsy that revealed residual mixed spindle cell/desmoplastic melanoma that was completely excised with negative margins and negative nodes (stage IIB, pT4A). Previous to this the patient had a history of Crohn’s disease requiring hospitalization and following lymph node dissection, treatment was changed from infliximab and azathioprine to single therapy vedolizumab, an inhibitor of integrin α4β7, with the intent of limiting immunosuppression as much as possible while still optimizing therapy for Crohn’s disease.

Surrounding Inflammatory Bowel Disease (IBD), the patient was initially diagnosed with Ulcerative Colitis in 1991 and did not require treatment until developing a perirectal abscess in 1999. At that time the diagnosis was changed to Crohn’s disease rather than Ulcerative Colitis. Crohn’s disease is heterogeneous in its clinical manifestations, and the Montreal classification schema is used to better categorize a patient’s clinical course by age of onset, disease location, and disease behavior. The patient’s Montreal classification was A2 (onset between 17 and 40 years old), L3 (ileocolon location) and B3p (penetrating behavior with perianal disease). He has had no extra-intestinal manifestations of his IBD. Following the perirectal abscess in 1999 the patient was started on mesalamine and had approximately yearly flares requiring prednisone tapers for disease control. In 2010 he required more frequent tapers and his symptoms began to more aggressively emerge if his prednisone dose was reduced below 20 mg daily. In 2011 he presented to our institution’s Gastroenterology clinic. Pathology review from the outside hospital colonoscopy biopsies in 2010 showed inflammation of the cecum, descending colon, sigmoid colon, and rectum consistent with moderate to severe colitis. A repeat colonoscopy in 2011 confirmed active moderate-severe disease. He was started on azathioprine and mesalamine enemas/suppositories and continued on oral mesalamine. On this regimen he was able to be weaned off prednisone with symptom control. His course was complicated by the development of shingles, which required dose reduction of his azathioprine. He had a repeat colonoscopy in the June 2012 that showed active disease in the terminal ileum, cecum, and right colon. He was then started on infliximab infusions every 8 weeks and continued on dose-reduced azathioprine and rectal mesalamine (enemas/suppositories). Repeat colonoscopy in June 2013 showed normal terminal ileum with mild colitis proximally and mild to moderate proctitis. His rectal mesalamine therapy was escalated and repeat colonoscopy in November 2015 showed Crohn’s disease to be in remission.

**Fig. 1:**
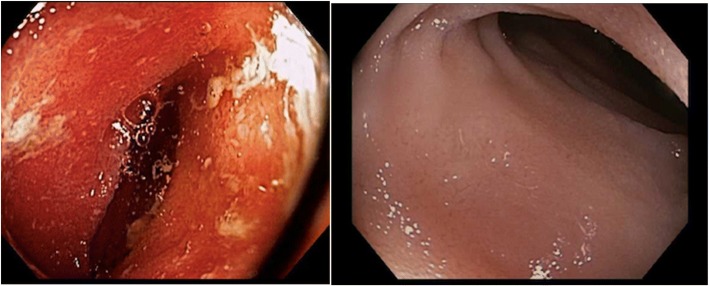
Comparison of Terminal Ileum from Colonoscopy in 2012 to Colonoscopy in 2017, (Left) inflammation of the terminal ileum on colonoscopy in 2012 while on azathioprine and oral/rectal mesalamine. (Right) normal mucosa of the terminal ileum on colonoscopy in May 2017 while on vedolizumab

In April 2016 IBD therapy was changed from infliximab and azathioprine/mesalamine to vedolizumab in response to his melanoma diagnosis. Vedolizumab has been administered 10 mg/kg intravenously every eight weeks in conjunction with IV steroids since that time. The patient underwent surveillance colonoscopy as recently as May 2017 with pathology consistent with normal ileum and colon and patchy quiescent colitis in the sigmoid and rectum (Fig. 1).

In April 2016 the patient presented to our Oncology clinic for initial consultation surrounding the diagnosis of melanoma when following resection and work up revealed no evidence of disease. In July 2017 he presented with a nodule on his scalp, and biopsy demonstrated recurrence of melanoma (*BRAF* wildtype; *NF1, SF3B1, TERT, TP53* variants). Subsequent positron emission tomography showed a hypermetabolic and large lytic lesion in the sacrum as well as a fludeoxyglucose avid lesions in the thyroid and lung, consistent with metastatic melanoma.

**Fig. 2:**
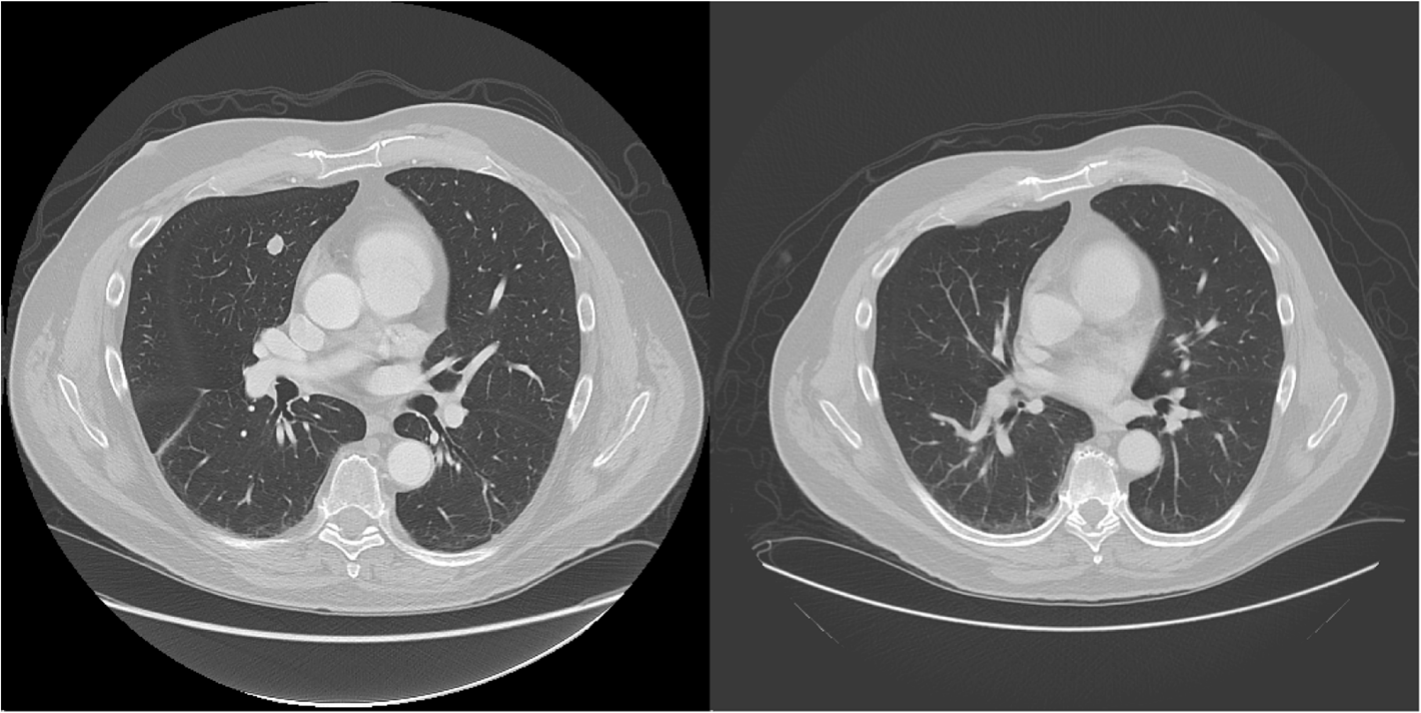
Resolution of Metastatic Pulmonary Nodule after Treatment with Pembrolizumab, **(**Left) CT chest from 9/22/2017 revealing RML nodule 1.2 × 1.0 cm in size. (Right) CT chest from 12/14/2017 demonstrating interval resolution of RML nodule after 4 cycles of pembrolizumab

**Fig. 3:**
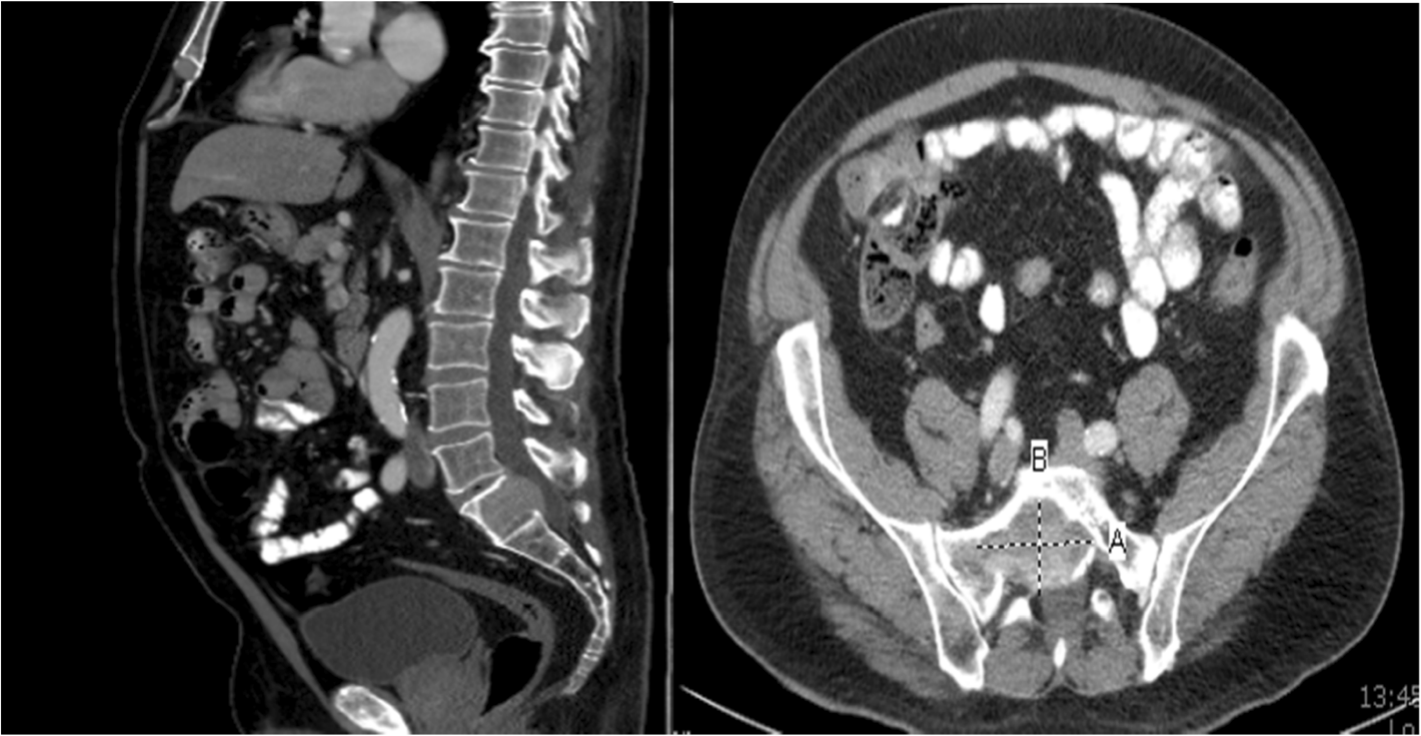
Comparison of Sacral Metastasis Showing No Progression after Treatment with Pembrolizumab and SBRT, (Left) CT abdomen/pelvis sagittal view from 9/22/2017 revealing metastasis in the S1 vertebral body extending posteriorly into the spinal canal. (Right) CT abdomen/pelvis axial view from 12/14/2017 showing unchanged size of sacral metastasis

Immunotherapy with pembrolizumab was initiated in September 2017. Additionally, stereotactic body radiation therapy (SBRT) was pursued for treatment of the large sacral mass including 22.5 Gy in three fractions. Of note the maximum cumulative dose of radiation his rectum could have received over the course of treatment is 50 cGy. Following the fourth cycle of pembrolizumab in December 2017, CT imaging revealed resolution of the previously visualized right middle lobe nodule (Fig. 2) and no growth of the sacral mass, consistent with our group’s published experience with pembrolizumab and SBRT (Fig. 3) [8]. Likely also contributing to this response is the abscopal effect from his radiation therapy as well as the synergistic effect of radiation therapy and immunotherapy which has been well-described in an Opinion article by Ngwa et al. [9] The patient now continues beyond cycle eleven of pembrolizumab with no evidence of progression of disease and having had no flare of IBD symptoms or toxicity related to radiation.

## Discussion and conclusion

Immune checkpoint blockade relies on pre-existent immunity to reinvigorate cancer surveillance and destruction. While checkpoint blockade demonstrates efficacy across many cancers, it can be accompanied by the untoward effects of immune-activation beyond the tumor in the form of irAEs. This inflammatory-mediated destruction occurs in the small and large bowels, lungs, endocrine glands and elsewhere. Thus, its use in patients with pre-existing autoimmune diseases has been limited. While not represented in registrational clinical trial programs, the use of checkpoint blockade in populations with autoimmunity or previous severe immunotherapy toxicity have been reported. Relevant to colitis; Johnson et al. published a retrospective review that included six patients with IBD who received ipilimumab for treatment of advanced melanoma [10]. Three of these patients had significant IBD requiring colectomies in the past and three were on either aminosalicylate or topical hydrocortisone. Two out of six had treatment-associated enterocolitis, successfully managed with either infliximab or methylprednisolone, while the other four had no flare or irAE. In another retrospective review [11], Menzies et al. included 119 patients with either underlying autoimmune disease (six with IBD) or major toxicity with ipilimumab who were being treated with anti-PD1 therapy (either pembrolizumab or nivolumab). None of the patients with IBD had a flare while on anti-PD1 therapy. However of the 52 total patients with autoimmune disorders, 38% developed a disease flare, and there was a trend for increased number of flares in patients who required immunosuppressive therapy at baseline for management of their autoimmune disorder. Leonardi et al conducted a retrospective review of 56 patients with non-small-cell lung cancer and concurrent autoimmune disease who received monotherapy with a PD-1/PD-L1 inhibitor [7]. A minority (13%) developed an exacerbation of their underlying disease while in total 55% experienced an exacerbation and/or unique irAE. None of the patients who experienced a disease flare required permanent discontinuation of immunotherapy while 11% of patients who experienced an irAE did. Also noted was the trend that patients who were symptomatic from their autoimmune disease at baseline were more likely to experience a disease flare when placed on anti-PD-1/PD-L1 therapy.

Of relevance to our patient, there is a published case series report of 7 patients in whom vedolizumab was used in the treatment of immune-related enterocolitis due to checkpoint blockade, including prophylactic use in one patient with pre-existing IBD who was being treated with ipilimumab [12]. However this patient went on to develop an IBD flare, bringing into question the utility of maintenance vedolizumab therapy in the setting of checkpoint blockade. Of note this patient differed from our patient in two important ways: 1) he had mildly active IBD disease prior to starting on vedolizumab and ipilimumab and 2) he was treated with anti-CTLA therapy, which has been shown to result in more frequent GI-related irAE than anti-PD-1 therapy [13].

We present a unique case of a patient who is maintained on vedolizumab with continued remission of his IBD while simultaneously being successfully treated with pembrolizumab for metastatic melanoma. Insight into how these potentially conflicting therapies interact may be informed by reviewing the biology of integrins and mechanism of anti-integrin therapy.

Integrins are transmembrane proteins located on leukocytes that facilitate migration from intravascular spaces to sites of tissue injury or inflammation. Integrins are essential to the establishment of an inflamed tissue microenvironment, allowing for the immune system to carry out its pathogen- and cancer-fighting responsibilities. However, it is this same pathway that also leads to the initiation and maintenance of inflammation in autoimmune conditions [14]. Thus, inhibition of integrin action perhaps can help suppress an overactive immune system. The first integrin inhibitor to be FDA approved for the treatment of Crohn’s disease was natalizumab, which was also used in the treatment of multiple sclerosis. Natalizumab blocks α4β1and α4β7 integrins on leukocytes from binding to intercellular adhesion molecules on endothelium cells. A rare but significant risk of natalizumab is the development of progressive multifocal leukoencephalopathy, a demyelinating disease caused by reactivation of the JC polyomavirus. The mechanism of this adverse effect is thought to be lack of immune system surveillance of the CNS due to blockade of α4β1, which plays an important role in localizing leukocytes to the brain. Vedolizumab is also an α4 inhibitor, however it only acts on α4β7. The integrin α4β7 provides an intestine-specific homing signal to leukocytes by binding to mucosal vascular addressin cell adhesion molecule 1 (MAdCAM-1), which is selectively expressed on mucosal endothelial cells of the gut. Thus, blockade of α4β7 results in gut-specific immunosuppression and has proven to be beneficial in the treatment of Crohn’s disease [15]. This ability to regulate leukocyte traffic in the bowel and not elsewhere is of obvious importance in the treatment of inflammatory bowel disease but at the same time also makes vedolizumab uniquely attractive when activation of the immune system elsewhere is desired. Our patient above was transitioned from his previous regimen of azathioprine and infliximab to vedolizumab upon his initial diagnosis of melanoma, with the intent of reducing the level of systemic immunosuppression in recognition of the immune’s system protective role in melanoma [16].

The idea of targeted immunosuppression in the gut to avoid checkpoint blockade induced enterocolitis has been investigated previously. Weber et al conducted a double-blind, placebo-controlled RCT using oral budesonide prophylactically in patients receiving ipilimumab for advanced melanoma [17]. Of note, patients with autoimmune diseases were excluded from the study. The results revealed that budesonide did not affect the rate of grade 2 or above diarrhea.

The presence of active symptoms of autoimmune disease and the use of immunosuppression therapy at the onset of ICPI treatment has been evaluated previously although with conflicting results. It seems reasonable to assume that patients with active symptoms of an autoimmune disease who are started on ICPI therapy would be more likely to experience a disease flare or irAE, and the retrospective review by Menzies et al. mentioned above supports this assumption with a statistically significant increased number of disease flares in patients with active symptoms vs clinically inactive disease. However, in a systematic review by Abdel-Wahab et al. looking at 123 patients from original case reports, case series, and observational studies, there was no difference in the frequency of adverse events in patients with active preexisting autoimmune disease [18]. Even less straightforward is whether or not baseline immunosuppression for autoimmune disease will be protective from a flare or irAE when ICPI therapy is initiated or a predictor of an adverse event. The study by Abdel-Wahab et al. revealed fewer adverse events in patients on immunosuppression prior to starting ICPI therapy whereas the Menzies et al. study demonstrated the opposite. Our patient had clinically inactive disease and was on immunosuppression prior to starting ICPI therapy, but it is clear that larger studies are needed in this group of patients to better elucidate these trends.

In our patient the concurrent use of vedolizumab, which selectively blocks T-cell migration into intestinal tissue, and pembrolizumab, which relies on cytotoxic T-cells already present in the tumor microenvironment to enact its effect, has so far resulted in continued suppression of his pre-existing IBD as well as complete regression of metastatic melanoma. Given the expected increase in use of checkpoint blocking immunotherapy in the future and the prevalence of autoimmune disease in the general population, the question of how to wield the immune system effectively will be of increasingly greater importance. This case suggests that with appropriately targeted immunotherapies, a patient with a pre-existing autoimmune condition can continue with immunosuppression while also receiving immune checkpoint inhibitor therapy.

## Abbreviations

IBD: Inflammatory bowel disease
ICPI: Immune checkpoint inhibitor
irAE: Immune related adverse event
MAdCAM-1: Mucosal vascular addressin cell adhesion molecule 1
PD-1/PD-L1: Anti-programmed death receptor-1/ligand
SBRT: Stereotactic body radiation therapy
